# Synchronous Germinal Center Onset Impacts the Efficiency of Antibody Responses

**DOI:** 10.3389/fimmu.2019.02116

**Published:** 2019-09-06

**Authors:** Theinmozhi Arulraj, Sebastian C. Binder, Philippe A. Robert, Michael Meyer-Hermann

**Affiliations:** ^1^Department of Systems Immunology, Braunschweig Integrated Centre of Systems Biology, Helmholtz Centre for Infection Research, Braunschweig, Germany; ^2^Centre for Individualized Infection Medicine (CIIM), Hanover, Germany; ^3^Institute for Biochemistry, Biotechnology and Bioinformatics, Technische Universität Braunschweig, Braunschweig, Germany

**Keywords:** germinal center, antibody production, vaccination, computer simulation, mathematical modeling

## Abstract

The germinal center reaction is an important target for modulating antibody responses. Antibody production from germinal centers is regulated by a negative feedback mechanism termed antibody feedback. By imposing antibody feedback, germinal centers can interact and regulate the output of other germinal centers. Using an agent-based model of the germinal center reaction, we studied the impact of antibody feedback on kinetics and efficiency of a germinal center. Our simulations predict that high feedback of antibodies from germinal centers reduces the production of plasma cells and subsequently the efficiency of the germinal center reaction by promoting earlier termination. Affinity maturation is only weakly improved by increased antibody feedback and ultimately interrupted because of premature termination of the reaction. The model predicts that the asynchronous onset and changes in number of germinal centers could alter the efficiency of antibody response due to changes in feedback by soluble antibodies. Consequently, late initialized germinal centers have a compromised output due to higher antibody feedback from the germinal centers formed earlier. The results demonstrate potential effects of germinal center intercommunication and highlight the importance of understanding germinal center interactions for optimizing the antibody response, in particular, in the elderly and in the context of vaccination.

## Introduction

Induction of an appropriate antibody response is critical for humoral immunity and efficient pathogen clearance. Vaccination relies primarily on modulating antibody responses and generating immune memory to boost the immune system against pathogens (1–4). T cell dependent antibody responses are mediated by germinal centers (GCs) where high-affinity plasma cells are formed starting from B cells with relatively lower affinities. During the GC reaction, the B cell receptor (BCR) is diversified by somatic hypermutation, followed by selection of B cells with higher affinity BCRs. B cell selection in GCs is T-cell mediated, where B cells with higher affinity BCR capture higher amounts of antigen from follicular dendritic cells (FDCs) and present the processed antigen to the T cells through pMHC (5–7). Altering the GC reaction is a promising way to modulate antibody responses (8). Injection of soluble antigen impacts the apoptosis and affinity maturation in the GCs (9, 10). Extended antigen availability has been shown to enhance the GC response (11, 12). Further, computational simulations have shown that extended antigen dosing can increase the GC response by increasing antigen capture (13).

Antibodies enhance or suppress antibody responses and mechanisms governing this are actively being studied (14, 15). Masking of antigen epitope by soluble antibodies is one of the suppressive mechanisms that has long been recognized (16). Bergström et al. have shown that administration of IgG suppresses extrafollicular antibody secreting cells, GC B cells, long-term plasma cells, IgG responses, and induction of memory response and demonstrated that antigen clearance is unlikely to be the mechanism underlying the observed effects (17). Epitope specificity of antibody response suppressed by injected antibodies are also observed (17, 18).

Injected antibodies are found to be deposited on FDCs and alter the apoptosis and affinity maturation of B cells in the GCs (19). Hence, a mechanism of self-regulation of GCs by the antibodies produced from plasma cells is proposed. Antibody feedback modulates antigen availability indirectly by masking antigen on FDCs and thus compete with the B cells for the antigen displayed on the surface of FDCs with dynamics determined by GC output (19). *In silico* analysis and simulations predicted that the injection of soluble antibodies promotes proper shutdown of the GC reactions and quicker affinity maturation due to increased selection efficiency (19). This also suggests that selection of B cells in the GCs could be influenced by the intercommunication between GCs due to soluble antibodies (19). Effects of altering antigen availability and Tfh help are also being studied extensively in the context of developing broadly neutralizing antibodies (12, 20).

Mathematical models are being developed and employed for identifying and understanding the mechanisms of many non-intuitive biological processes (21, 22). *In silico* simulations have facilitated a better understanding of the B cell-T cell interactions in spleen (23), GC reaction and interpretation of experimental results concerning GC kinetics, affinity maturation and antibody production (13, 20, 24–30).

Understanding the mechanisms that regulate antibody responses is important for devising specific optimization strategies to improve the vaccination response. The effects of GC-GC interactions due to soluble antibodies on individual GC reactions are not known. Here, we focus on understanding the contribution of interaction between GCs in affecting the antibody responses by extending a previously developed agent-based model of the GC reaction to cover inter-GC interaction and related read-outs (see Materials and methods). We study the impact of antibody feedback on shutdown and affinity maturation of GC reactions by varying the strength of antibody feedback. We also investigate the effect of antibody feedback when the GC onset is delayed after the onset of earlier GCs already producing antibody. We propose that a change in the number of germinal centers and asynchronous GC initiation could have implications in GC function due to altered antibody feedback.

## Materials and Methods

Cells are represented as agents on a three-dimensional lattice with a lattice constant of 5 μm. The GC reaction volume is a sphere of radius 160 μm within the lattice and is divided equally into dark and light zones. Founder B cells enter the dark zone at a rate of 2 cells/h and divide six times (30). Dividing centroblasts mutate at a probability of 0.5 starting from day 1 of the reaction. Centroblasts differentiating to centrocytes search for antigen on FDCs and their successful contact depends on the BCR affinity. A four-dimensional shape space is used for affinity representation (31). B cells that failed to collect antigen within the collection period undergo apoptosis. Further, Tfh signaling is polarized toward the B cell that collected the maximum amount of antigen, thus, preferentially selecting high affinity B cells which were more efficient in collecting and processing antigen. Selected cells at the border of the reaction volume exit toward the T zone and differentiate into antibody producing plasma cells at a rate of $\frac{\ln\;2}{24}h^{-1}$. An agent-based representation was chosen in order to represent the behavior of individual cells and their interactions with a high degree of accuracy and the model has been adapted (26, 29) to be consistent with experimental findings on spatial dynamics, selection (32), and evolution of clonal dominance (33). A detailed description of the model is provided in the Supplementary Material.

### Antibody Production and Feedback

Antibodies are resolved into 11 bins (*i* = 0, 1, …, 10) reflecting their affinities. Change in the concentration of antibody *A*(*i*) in each bin *i* follows the equation:

(1) $$\begin{array}{r} \frac{dA\left(i\right)}{dt}=k_{1}n_{p}\left(i\right)-k_{2}A\left(i\right) \end{array}$$

The concentration of antibody in each bin is increased at a rate *k*_1_, reflecting the production of antibodies with 10^−17^ mol/h from each plasma cell (*n*_*p*_(*i*) is the number of plasma cells with affinity corresponding to bin *i*) and their dilution over a volume of 10 ml. Hence, the total antibody produced is diluted over the whole organism and the concentration is assumed to be the same everywhere. Antibodies have a half-life of 30 days ($k_{2}=\frac{\ln\;2}{30}day^{-\;1}$).

Antibodies attributed to the different bins have the same association rate constant *k*_*on*_ (10^6^ M^−1^.s^−1^) (34), while the *k*_*off*_ varies such that their dissociation constants are between 10^−5.5^ and 10^−9.5^ M. These antibodies form immune complex (*C*_*FDC*_) with antigen displayed on each FDC site (*G*_*FDC*_) following the dynamic equation:

(2) $$\begin{array}{r} \frac{dC_{FDC}\left(i\right)}{dt}=k_{on}G_{FDC}\left(NA\left(i\right)+A_{early}\left(i\right)\right)-k_{off}\left(i\right)C_{FDC}\left(i\right) \end{array}$$

Here, *A*(*i*) is the antibodies produced by the simulated GC and *A*_*early*_(*i*) is the antibodies produced by early GC used in the simulations of delayed initialization of GC (see methods–Simulation of delayed initialization of GCs). *N* is a scaling factor controlling the strength of antibody feedback onto the simulated GC. We assume that GCs throughout the organism concurrently produce antibodies which are homogeneously distributed on the whole organism and in particular appear in the simulated GC.

We assume that the soluble antibodies reversibly bind to FDC antigen and bound antigen is not available for uptake by B cells. This results in competition between B cells and soluble antibodies to bind to the antigen. With each successful contact with FDCs, B cells consume an antigen portion equivalent to 10^−8^ M. The amount of free antigen decreases or increases due to immune complex formation or dissociation. The decrease in the concentration of antibodies due to immune complex formation is neglected.

### Immune Power Calculation

A measure for the efficiency of a GC reaction termed *immune power* (IP) is introduced (35), which reflects a combination of affinity maturation and the amount of produced antibody forming plasma cells. The IP estimates the ability of antibodies produced by a GC in binding the antigen. For this, we use an antigen concentration (G) of 10^−6^ M and estimate the proportion of antigen bound to the antibodies of a particular affinity. We assume a high concentration of antigen when compared to the concentration of antibodies and use the following steady state approximation to calculate the concentration of bound antigen:

(3) $$\begin{array}{r} G_{bound}\left(i\right)=\frac{A\left(i\right)G}{K\left(i\right)+G} \end{array}$$

where *i* is the bin number, *A*(*i*) is the concentration of antibody produced by the simulated GC and *K*(*i*) = 10^−5.5−0.4*i*^ is the dissociation constant. IP is calculated as the ratio of antigen bound to the soluble antibodies to the total antigen:

(4) $$\begin{array}{r} IP=\frac{\sum_{i}G_{bound}\left(i\right)}{G} \end{array}$$

IP combines the effects of changes in quality (affinity) as well as quantity (concentration) of the antibodies produced in binding the antigen.

### Simulation of Delayed Initialization of GCs

To simulate a GC initialized with a certain delay (with respect to earlier initialized GC), we simulate this GC reaction (*N* = 1) along with externally added antibodies *A*_*early*_ (see Equation 2) at every time step. The added antibodies correspond to soluble antibodies produced from earlier initialized GC. For this, we generate an antibody concentration profile of a GC initialized first and rescale it with a scaling factor of 300 to reflect an antibody concentration for strong feedback. Delay of initialization of the simulated GC is achieved by adding the externally added antibodies after shifting the antibody concentration profile depending on the time of delay. Hence, the GC initialized late is under the influence of antibody feedback due to earlier initialized GCs. Feedback of antibodies produced from the late GC on early GC is ignored.

## Results

We use the previously described agent-based model to test the influence of antibody feedback on the output of the GC reaction with a focus on the termination of the GC reaction and affinity maturation of B cells. We vary the scaling factor (*N*) for antibody production as a proxy to varying the strength of antibody feedback. Increasing N is similar to increasing the number of synchronous GCs producing antibodies although we are simulating a representative GC. Here, we use the scaling factors 1, 10, 30, and 300.

**Figure 1:**
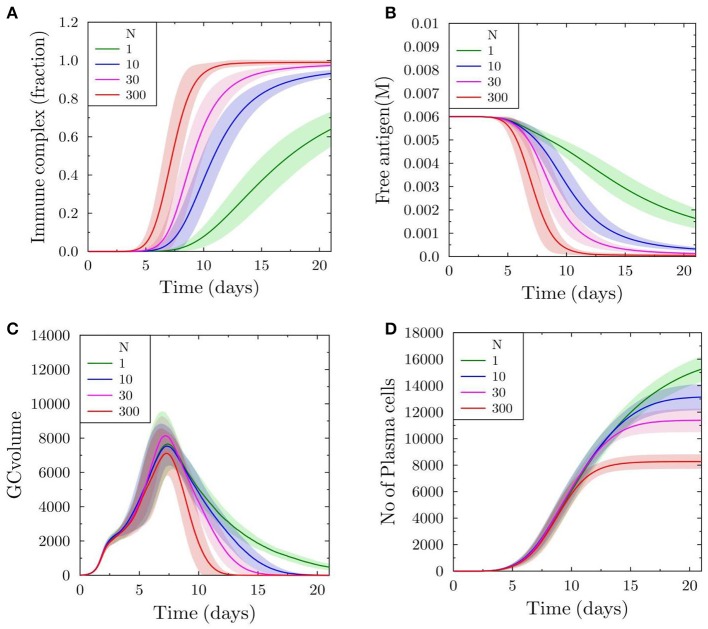
Germinal center reaction kinetics with varying antibody feedback strength. The scaling factor *N* is proportional to the strength of the antibody feedback onto the simulated GC. **(A)** Fraction of FDC antigen bound in immune complexes. **(B)** Concentration of free antigen. **(C)** GC volume kinetics measured as number of GC-BCs. **(D)** Total number of plasma cells generated in the simulated GC reaction over time.

To monitor the binding of soluble antibodies to antigen displayed on FDCs, we plot the fraction of total antigen on FDCs that is a part of immune complex formed with soluble antibodies. With scaling factor *N* = 1, approximately 65% of the total antigen is bound to soluble antibodies on day 21 (Figure 1A). However, as the feedback strength is increased (*N* = 10, 30, and 300), the proportion of immune complex increases more quickly and almost all antigen on FDCs is covered with antibodies at an earlier time point (Figure 1A). The proportion of antigen not bound to soluble antibodies is available for uptake by B cells searching for antigen on FDCs. Consequently, the concentration of free antigen available for B cells drops more quickly with increasing antibody feedback (Figure 1B), which increases the selection pressure for B cells.

In order to understand the progress and duration of the GC reaction, we plot the GC volume defined as the total numbers of centroblasts and centrocytes prior to the formation of output cells that are in turn capable of differentiating to antibody producing plasma cells. With increasing feedback, the GC volume reaches zero more quickly, suggesting that the termination is accelerated due to antibody feedback (Figure 1C). This is consistent with the results of previous studies (19). The number of plasma cells differentiated from output cells is higher with lower antibody feedback and it decreases markedly in the case of higher feedback strengths (Figure 1D). This observation is a consequence of the low antigen availability for B cells that increases its selection pressure resulting in the selection of fewer B cells.

**Figure 2:**
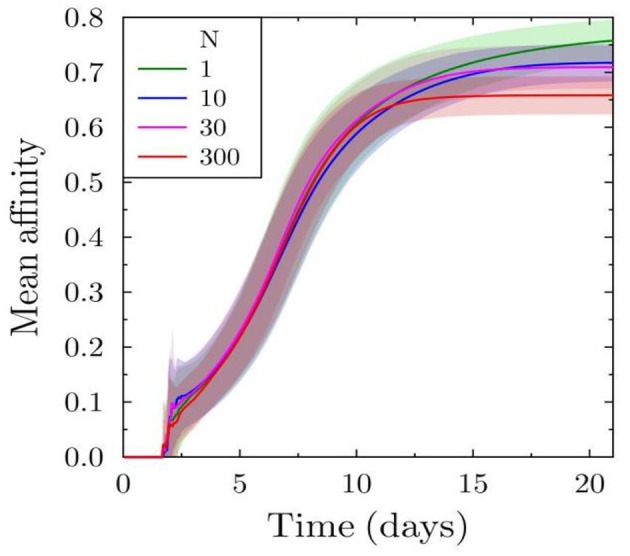
Effect of varying antibody feedback on affinity maturation: mean affinity of all plasma cells derived from the simulated GC reaction.

We further investigate the affinity maturation process, as this could also be a target due to the selection pressure induced by the decreased antigen availability for B cells. Approximately after 10 days, the affinity in the case of low antibody feedback is higher (Figure 2) but the effect is weak compared to that on the number of plasma cells produced. The lower mean affinity observed at later time points is a consequence of earlier shutdown. Hence, the earlier shutdown has decreased the time for effective affinity maturation and, thus, suppressing a further increase in affinity. This suggests that premature termination of the GC reaction would not only decrease the amount of selected output cells but could also decrease the effectiveness of the affinity maturation process.

**Figure 3:**
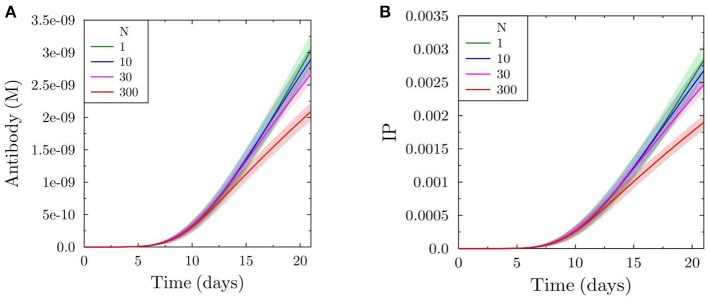
Total antibody produced by simulated GC **(A)** and efficiency of the GC reaction in terms of immune power (IP) (defined in Equation 4) **(B)**.

In order to quantitatively capture the product of both effects of increased antibody feedback, namely the intermediate change in affinity and the reduced production of output cells, we calculate the immune power IP (see Equation 4) by estimating the fraction of antigen bound to the antibodies produced from the plasma cells (Figure 3A). As expected from the relative strength of both effects, the immune power is reduced with increasing feedback strength, reflecting a decrease in the efficiency of the GC reaction (Figure 3B).

**Figure 4:**
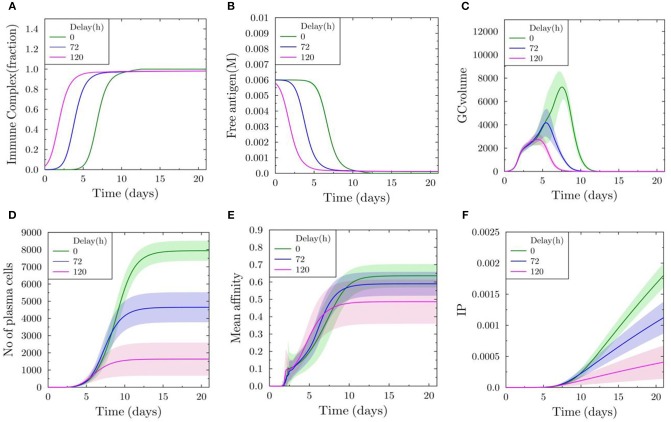
Delayed initialization of GC reaction (*N* = 1) with antibody feedback from early GCs (*N* = 300). **(A)** Fraction of FDC antigen bound to soluble antibodies. **(B)** Concentration of free antigen. **(C)** GC volume as number of GC B cells. **(D)** Number of all plasma cells produced in the delayed GC reaction. **(E)** Mean affinity of all plasma cells generated in the delayed GC reaction. **(F)** Immune power (IP) (defined in Equation 4) of the delayed GC reaction.

Next, we performed simulations of GC (*N* = 1) initialized with a delay of 0, 72, and 120 h after the initialization of GCs (*N* = 300) inducing antibody feedback. The antibody from the early GCs is used as an input for the simulation of the late GC. Without delay, the fraction of immune complexes on FDCs reaches 1 approximately in 10 days (Figure 4A). The concentration for free antigen available for B cells drops more quickly with increasing delay (Figure 4B). GC volume kinetics show a decrease in the maximum volume and earlier shutdown with increasing delay (Figure 4C). A GC initialized 120 h later is terminated even before 10 days and the maximum volume is reduced by 75% (Figure 4C). The number of plasma cells formed is dramatically reduced with increasing delay (Figure 4D). There is also a decrease in the mean affinity with increasing delay (Figure 4E), although there is a small increase observed at earlier time points prior to 10 days. The strong antibody feedback pushes affinity maturation of a few B cells, but mostly suppresses selection. Correspondingly, the immune power is reduced with delayed initialization, showing a decrease in the efficiency of late initialized GCs (Figure 4F).

**Figure 5:**
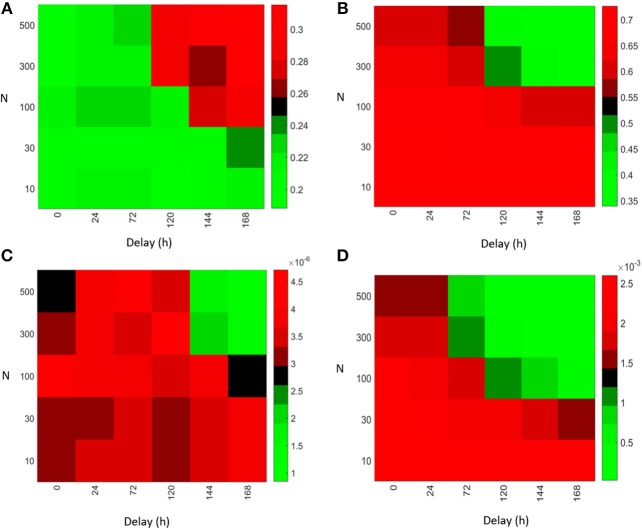
Initialization of a GC (*N* = 1) with varying delay with respect to early GCs simulated with different scaling factors *N*. Mean affinity of plasma cells on day 5 **(A)** and day 21 **(B)** after GC initialization. Immune power (IP) (defined in Equation 4) of the delayed GC on day 5 **(C)** and day 21 **(D)**.

In order to compare the importance of the strength of antibody feedback vs. GC delay on affinity maturation and output production, we systematically varied both parameters (see Figure 5). Increasing the value of either parameter increases the strength of the antibody feedback and can replace the effect of the respective other. The mean affinity of plasma cells on day 21 decreased from ~0.7–0.35 in the range of parameter values tested (Figure 5B). The increase in mean affinity observed on day 5 is very low (Figure 5A) compared to the decrease observed on day 21. Hence, affinity maturation is improved by antibody feedback early in the GC reaction but is not continued in the long term. In contrast, the immune power exhibits a consistent decrease with increasing antibody feedback strength or GC delay at any time of the GC reaction (Figures 5C,D). Hence, the efficiency of the GC reaction is dominated by the negative effect of antibody feedback on the number of plasma cells rather than the intermediate positive effect on the affinity of plasma cells.

## Discussion

Using an agent-based model approach, we investigated the role of soluble antibodies formed as a result of the GC reaction in modulating the output of the GCs. Our simulations with varying strength of antibody feedback show that the increased antibody feedback results in a decreased production of plasma cells. GC reactions terminate earlier with increased antibody feedback. Immune complex formation of FDC-bound antigen with soluble antibodies results in a decreased availability of antigen for the B cells undergoing selection. The raised selection pressure induces a decreased production of plasma cells and earlier termination of the GC.

Although the increased selection pressure is expected to improve or accelerate affinity maturation, we find that the improvement in affinity maturation by antibody feedback is weak. There is a small improvement in the affinity at earlier time points. However, because of the reduction of GC size kinetics, there is also a termination of the affinity maturation process. With higher antibody feedback, the affinity maturation process is prematurely terminated and, hence, the mean affinities of plasma cells do not improve further in later stages of the GC reaction. Therefore, the mean plasma cell affinity remains low when compared to lower antibody feedback, which allows for better affinity maturation. These observations suggest that kinetics of the GC reaction is the key process targeted by antibody feedback and the effect on affinity maturation is dominated by the earlier termination of the GC reaction.

Calculation of the efficiency of GC reactions (immune power) shows an overall decrease in the efficiency due to the decreased amount of antibodies (due to lower number of plasma cells) and a reduction in the mean affinity. This suggests that the increased selection pressure due to antibody feedback could have a detrimental effect on the functioning of the GC. Moreover, it suggests the role of soluble antibodies in regulating the GC reaction, preventing chronic continuation of GC reactions, and preventing the excess production of plasma cells under normal conditions. As the soluble antibodies are produced as the result of the GC reaction, antibody feedback might be considered as a natural mechanism of self-regulation of the production of antibodies and changes in their affinities.

Considering the interactions between multiple GCs, it is likely that similar scaling of antibody feedback might occur with increasing the number of GCs producing plasma cells. Consequently, the output of individual GCs might be altered depending on the number of GCs formed in response to immunization.

Further, our simulations show that delayed initialization of a GC reaction would decrease the production of plasma cells by impairing the GC size kinetics and decrease the extent of affinity maturation, resulting in a decreased efficiency due to antibody feedback from GCs initialized earlier. Asynchronous onset of GCs is reflected in experimental findings as the number of GCs seem to increase over a period of time after immunization (36, 37). This suggests that synchronization of GC onset can alter the efficiency of GCs.

The combination of the number of GCs and the extent of synchronization of their onset might play a role in determining the efficiency of overall antibody responses due to GC-GC interactions. The number of GCs formed in the elderly in response to immunization is reduced (36, 38). Such changes might result in alterations in antibody feedback in addition to other defects.

Future studies on understanding the number of GCs induced and asynchronous onset might allow precise prediction of changes in antibody responses and ways to modulate them. In this study, we focus mainly on the contribution of plasma cells to the antibody feedback. Hence, the effects of low affinity antibodies produced by extrafollicular antibody secreting cells on GC onset and its contribution to the antibody feedback are neglected. In the case of a secondary immune response, the antibody feedback on GCs might be increased due to a higher extrafollicular response generated by memory B cells. A large number of factors influence vaccination success including cytokine production, B and T repertoire diversity and proportion of T regulatory cells (39, 40). Several strategies have been suggested to improve vaccine efficacy including extended antigen availability, targeting pattern recognition receptors, dendritic cells, and T cells (11, 12, 41–44). In addition to increased Tfh help (11, 12) the enhanced GC response observed upon prolonged antigen delivery could also be an outcome of overcoming antibody feedback by increased antigen deposition on FDCs. Our results show that changes in antibody feedback due to GC-GC interactions might also play a role in the optimization of vaccination.

## Author Contributions

PR, SB, and MM-H designed the study. MM-H developed the model. TA performed the simulations. TA, SB, and MM-H analyzed the results and wrote the manuscript.

### Conflict of Interest Statement

The authors declare that the research was conducted in the absence of any commercial or financial relationships that could be construed as a potential conflict of interest.
