# Supplementary material for: Synchronous Germinal Center Onset Impacts the Efficiency of Antibody Responses
Source: Front Immunol. 2019 Sep 6;10:2116. doi: 10.3389/fimmu.2019.02116 (PMC6742702; doi:10.3389/fimmu.2019.02116)
Supplement: Supplementary file 1 [file Data_Sheet_1.pdf]

Supplementary Material

Germinal Centre simulation model

The GC model assumptions underlying the germinal centre (GC) simulations are explained here and include the used parameter values. It follows the description of [1] adapted to include novel features introduced since then in [2, 3], as well as the description of antibody production, feedback, and interaction with antigen presented on follicular dendritic cells. Used acronyms are: DZ for dark zone, LZ for light zone, Tfh for T follicular helper cell, FDC for follicular dendritic cell.

Space representation

All reactions take place on a three-dimensional discretized space with a rectangular lattice with lattice constant of $\Delta x = 5\mu m$. Every lattice node can be occupied by a single cell only.

Shape space for antibodies

Antibodies are represented on a four dimensional shape space [4]. The shape space is restricted to a size of 10 nodes per dimension, thus, only considering antibodies with a minimum affinity to the antigen. The optimal clone Φ^* is positioned in the centre of the shape space. A position on the shape space Φ is attributed to each B cell. Its Hamming distance $||\Phi - \Phi^*||_1$ to the optimal clone is used as a measure for the antigen binding probability. The binding probability is calculated from the Gaussian distribution with width $\Gamma = 2.8$ [5]:

$$b(\Phi, \Phi^*) = \exp\left(-\frac{||\Phi - \Phi^*||_1^2}{\Gamma^2}\right) \quad . \tag{1}$$

B cell phenotypes

Three B cell phenotypes are distinguished: DZ B cells, LZ B cells, and output cells. The different phenotypes characterize the cell properties and are not meant as localization within the GC zones. DZ B cells divide, mutate and migrate. LZ B cells also migrate and undergo the different stages of the selection process. Output cells only migrate.

Founder cells

The model starts from 250 Tfh, 200 FDCs, 300 stromal cells, and no B cell. Tfh are randomly distributed on the lattice and occupy a single node each. Stromal cells are restricted to the DZ (see section *Chemokine distribution* for their function). FDCs are restricted to the upper half of the reaction sphere, occupy one node by their soma and have 6 dendrites of $40\mu m$ length each. The presence of dendrites is represented as a lattice-node property and, thus, visible to B cells. The dendrites are treated as transparent for B cell or Tfh migration such that they do not inhibit cell motility.

B cell influx

We assumed that B cells enter the GC reaction with a probability corresponding to a rate of 2 cells per hour. New B cells are randomly positioned on the lattice (exclusively on free nodes). The shape space position of each new B cell is randomly picked from a set of 100 shape space positions anywhere in the shape space.

Antigen-presentation by FDCs

Each FDC is loaded with 3000 antigen portions distributed onto the lattice-nodes occupied by FDC-soma or FDC-dendrite. One antigen portion corresponds to the number of antigen molecules taken up by a B cell upon successful contact with an FDC.

Antigen-antibody interaction on FDCs

The source of antibodies are output cells of the GC reaction (see section *Antibodies* below). Antibodies are represented in the 4-dimensional shape space with 10 positions in each direction. The quantity of interest is the amount of free antigen at each FDC site when antibodies are present and changing over time. As it is not feasible to calculate the amount of free antigen at each site for all 10,000 possible antibody types, 11 affinity bins $A(i)$ with $i \in [0, \ldots, 10]$ were introduced, where the affinity is defined relative to the antigen. We assumed a constant on-rate $k_{\mathrm{on}} = 10^6/(\mathrm{Mol\,sec})$ [6] and a variable off-rate

$$k_{\mathrm{off,i}} = \frac{k_{\mathrm{on}}}{10^{5.5+0.4i}} \quad , \tag{2}$$

reflecting a dissociation constant that varies over 4 orders of magnitude. At each FDC site x, the chemical kinetics equation for the immune complexes $C_{\mathrm{FDC}}(i,x)$ formed between antibodies in bin i and antigen at the FDC site x

$$\frac{dC_{\mathrm{FDC}}(i,x)}{dt} = k_{\mathrm{on}} G_{\mathrm{FDC}}(x) A(i) - k_{\mathrm{off}}(i) C_{\mathrm{FDC}}(i,x) \tag{3}$$

was solved in order to determine the amount of free antigen $G_{\mathrm{FDC}}(x)$ at this site. Only this amount of antigen is available for B cells to bind antigen with probability according to Eq. (1).

Note that for variation of the strength of antibody feedback $A(i)$ was replaced by

$$A(i) \rightarrow NA(i) + A_{\mathrm{early}}(i) \ , \tag{4}$$

where N is a factor scaling the strength of antibody feedback. $A_{\mathrm{early}}(i)$ is the amount of antibodies generated by earlier GCs which is used for simulations of delayed GCs together with $N = 1$ and is zero otherwise.

Antibodies

Output cells from the GC reaction are recollected and memorized together with the affinity of the encoded antibody to the antigen. Their life time is assumed longer than the duration of the GC reaction. Output cells are attributed to the different affinity bins for each antigen and further differentiate to an antibody forming plasma cell according to a linear rate equation with a rate of $\ln(2)$ per day, i.e. with a half life of one day.

All plasma cells produce antibodies $A(i)$, attributed to bin i according to

$$\frac{dA(i)}{dt} = k_1 n_{\mathrm{P}}(i) - k_2 A(i) \ , \tag{5}$$

where k_1 is a production rate per cell of $10^{-17}\mathrm{mol/hour}$ [7] spread over a volume of $10\,\mathrm{ml}$. The produced antibodies are assumed to quickly distribute over the whole organism and, in particular, are homogeneously distributed over the space of the simulated GC. $k_2 = \ln(2)/(30\mathrm{days})$ is the degradation rate of antibodies.

DZ B cell division

The average cell cycle duration of 7 hours of DZ B cells is varied for each B cell according to a Gaussian distribution. This is needed to get desynchronization of B cell division. The cell cycle is decomposed into four phases (G1, S, G2, M) in order to localize mitotic events if this is needed.

Each founder B cell divides a number of times before differentiating to the LZ phenotype for the first time. Six divisions was the number of divisions found in response to the extreme stimulus with anti-DEC205-OVA [8, 1]. Each selected B cell divides an number of times determined by the interaction with Tfh (see below, LZ B cell selection). The parameters of the interaction with Tfh are tuned such that the mean number of divisions is in the range of two [9]. This value is required in order to maintain a DZ to LZ ratio in the range of two [8, 1].

A division requires free space on one of the Moore neighbors of the dividing cell. Otherwise the division is postponed until a free Moore neighbor is available.

At every division the encoded antibody can mutate with a probability of 0.5 [10, 11]. This corresponds to a shift in the shape space to a von Neumann neighbor in a random direction. Upon selection by Tfh the mutation probability is individually reduced from $m_{\max} = 0.5$ down to $m_{\min} = 0$ in an affinity-dependent way following

$$m(b) = m_{\max} - (m_{\max} - m_{\min})\, b \tag{6}$$

with b from Eq. (1) [12]. Thus, after recycling DZ B cells can acquire reduced mutation probabilities. This mechanism is motivated by the observation that B cell receptor internalization enhances the activation of the kinase Akt [13] which, in turn, suppresses activation induced cytosine deaminase (AID) [14]. AID is required for somatic hypermutation, such that this provides an affinity-dependent down-regulation of the mutation frequency [15]. However, there is no formal proof of this mechanism.

B cell division of B cells that previously acquired antigen and have been selected by Tfh distribute the retained antigen asymmetrically to the daughters [16]. The model assumes asymmetric division in 72% of the cases, which is supported by experimental observations (see [16] and Supplementary Figure S1 in [1]). If division is asymmetric, one daughter gets all the retained antigen while the other gets none, which approximates the value of 88% found in [16]. Mutation is suppressed in asymmetric divisions, which is an arbitrary choice.

After the required number of divisions the B cell differentiates with a rate of in $1/6$ minutes to the LZ phenotype. All B cells that kept the antigen up to this time, differentiate to output cells, up-regulate CXCR4, and leave the GC in direction of the T zone. The alternatives, that B cells randomly differentiate to output cells after divisions with a probability of 23% (LEDAX model in [1]) or that B cells decide to differentiate to output cells right after interaction with Tfh (BASE mode in [1]), leads to very similar GC readouts. However, the amount of generated

output cells is substantially higher if the B cells differentiate to output cells after divisions as compared to after selection [15].

LZ B cell selection

LZ B cells can be in the states *unselected, FDC-contact, FDC-selected, Tfh-contact, selected, apoptotic*.

Unselected

LZ B cells migrate and search for contact with FDCs loaded with antigen in order to collect antigen for 0.7 hours. If an FDC soma or dendrite is present at the position of the B cell, the B cell attempts to establish contact to the antigen. Binding is affinity dependent and happens with the probability b in Eq. (1). If the available number of antigen portions at the specific FDC site drops below 20 the binding probability b is linearly reduced with the number of available portions. If successful, the B cell switches to the state *FDC-contact*; otherwise the B cell continues to migrate. Further binding-attempts are prohibited for 1.2 minutes. At the end of the antigen collection period, B cells switch to the state *FDC-selected*. If a LZ B cell fails to collect any antigen at this time it switches to the state *apoptotic*.

FDC-contact

LZ B cells remain immobile (bound) for 3 minutes [17] and then return to the state *unselected*. The counter for the number of successful antigen uptake events is increased by one and the FDC reduces its locally available antigen portions by one.

FDC-selected

B cells search for contact with Tfh. If they meet a Tfh they switch to the state *TC-contact*.

Tfh-contact

LZ B cells remain immobile for 36 minutes. In this time the bound Tfh, which may also be bound to other B cells, polarizes to the bound B cell with highest number of successful antigen uptakes. Only this B cell receives Tfh signals and accumulates those. After the binding time, the B cell switches to the state *apoptotic* if the Tfh was polarized for less than 30 minutes to it. Otherwise it switches to the state *selected*. Note that other variants of the mode exist in which a B cell interacts with multiple Tfh for shorter times ([18, 19]) and collect signals from these interactions before fate decision.

Selected

LZ B cells keep the LZ phenotype for six hours and desensitize for CXCL13, thus, perform a random walk. During that time they re-enter cell cycle and progress through the cell cycle phases. Then they recycle back to the DZ phenotype with a rate of $1/6$ minutes and memorize the amount of collected antigen as well as the cell cycle phase they have achieved by this time.

The number of divisions $P(A)$ the recycled B cells will do is derived from the amount of collected antigen A, which reflects the amount of pMHC presented to Tfh and the affinity of the

B cell receptor for the antigen, as follows:

$$P(A) \;=\; P_{\text{min}} + (P_{\text{max}} - P_{\text{min}})\,\frac{A^{n_{\text{P}}}}{A^{n_{\text{P}}} + K_{\text{P}}^{n_{\text{P}}}}\;. \qquad (7)$$

The more antigen was collected by the B cell, the more divisions are induced. We set the minimum number of division to one ($P_{\text{min}} = 1$) in order to avoid recycling events without further division. It is limited by six divisions in the best case, which is motivated by anti-DEC205-OVA experiments in which DEC205$^{+/+}$ B cells received abundant antigen which increased pMHC presentation to a maximum [8]. The population dynamics in vivo and in silico only matched when the number of divisions was increased to six in the simulation [1] suggesting that the strongest possible pMHC presentation to Tfh induces six divisions ($P_{\text{max}} = 6$). The Hill-coefficient was set to $n_{\text{P}} = 2$.

The half value K_{P} remained to be determined, which denotes the amount of antigen collected by B cells at which the number of divisions becomes half maximal. The number of collected antigen portions varies between zero and a maximum determined by the duration of the antigen collection phase, the duration of each B cell interaction with FDCs, and the migration time between two antigen presenting sites. The numbers of successful B cell-FDC encounters as observed in the simulations served as estimate of A_{max}. Low affinity B cells had zero or one antigen uptake event, while high affinity cells took up between 5 and 10 portions. For an intermediate antigen uptake of $A_0 = 4.5$, the resulting number of divisions has to be $P_0 = 2$ in order to be in agreement with the mean number of divisions in the range of two [9], which leads to the condition:

$$K_{\text{P}} \;\approx\; A_0 \left(\frac{P_{\text{max}} - P_{\text{min}}}{P_0 - P_{\text{min}}} - 1\right)^{1/n_{\text{P}}} \;=\; 9 \quad . \qquad (8)$$

Apoptotic

LZ B cells remain on the lattice for 6 hours before they are deleted. They continue to be sensitive to CXCL13 during this time.

Chemokine distribution

Two chemokines CXCL12 and CXCL13 are considered. CXCL13 is produced by FDCs in the LZ with 10nMol per hour and FDC while CXCL12 is produced by stromal cells in the DZ with 400nMol per hour and stromal cell. As both cell types are assumed to be immobile, chemokine distributions were pre-calculated once and the resulting steady state distributions were used in all simulations.

Chemotaxis

DZ and LZ B cells regulate their sensitivity to CXCL13 and CXCL12, respectively. This is true in all B cell states unless stated otherwise. All B cells move with a target speed of $7.5\mu m/min$. This leads to a slightly lower observable average speed of $\approx 6\mu m/min$.

B cells have a polarity vector that determines their preferential direction of migration. The polarity vector $\vec{p}$ is reset every 1.5 minutes into a new direction using the chemokine distribution c as

$$\vec{p} \;=\; \vec{p}_{\text{rand}} + \frac{\alpha}{1 + \exp\left\{\kappa\left(K_{1/2} - \Delta x|\vec{\nabla}c|\right)\right\}}\,\frac{\vec{\nabla}c}{|\vec{\nabla}c|} \quad , \qquad (9)$$

where $\vec{p}_{\rm rand}$ is a random polarity vector and the turning angle is sampled from the measured turning angle distribution ([20] Fig. S1B). $\alpha = 10$ determines the relative weight of the chemotaxis and random walk, $K_{1/2} = 2 \cdot 10^{11}$ Mol determines the gradient of half maximum chemotaxis weight, and $\kappa = 10^{10}/\mathrm{Mol}$ determines the steepness of the weight increase.

B cells de- and re-sensitize for their respective chemokine depending on the local chemokine concentration: The desensitization threshold is set to 6nMol and 0.08nMol for CXCL12 and CXCL13, respectively, which avoids cell clustering in the center of the zones. The resensitization threshold is set at $2/3$ and $3/4$ of the desensitization threshold for CXCL12 and CXCL13, respectively.

B cells can only migrate if the target node is free. If occupied and the neighbor cell is to migrate in the opposite direction (negative scalar product of the polarity vectors) both cells are exchanged with a probability of 0.5. This exchange algorithm avoids lattice artifacts leading to cell clusters.

Tfh do random walk with a preferential directionality to the LZ: The polarity vector $\vec{p}$ of Tfh is determined from a mixture of random walk $\vec{r}$ and the direction of the LZ $\vec{n}$ by

$$\vec{p} \;=\; (1-\alpha')\vec{r} + \alpha'\vec{n}\,, \tag{10}$$

where $\alpha' = 0.1$ is the weight of chemotaxis. This weight leads to a dominance of random walk with a tendency to accumulate in the LZ as found in experiment. TCs migrate with an average speed of $10\mu m/min$ and repolarize every 1.7 minutes [21].

Output cell motility is derived from plasma cell motility data to be $3\mu m/min$ [20] with a persistence time of 0.75 minutes.

References

1. Meyer-Hermann, M., Mohr, E., Pelletier, N., Zhang, Y., Victora, G. D., and Toellner, K.-M. A theory of germinal center B cell selection, division, and exit. *Cell Rep.* **2**, 162–174 (2012).

2. Meyer-Hermann, M. Overcoming the dichotomy of quantity and quality in antibody responses. *J. Immunol.* **193**, 5414–5419 (2014).

3. Binder, S. and Meyer-Hermann, M. Implications of intravital imaging of murine germinal centers on the control of B cell selection and division. *Front. Immunol.* **7**, 593 (2016).

4. Perelson, A. S. and Oster, G. F. Theoretical Studies of Clonal Selection: Minimal Antibody Repertoire Size and Reliability of Self-Non-self Discrimination. *J. Theor. Biol.* **81**, 645–670 (1979).

5. Meyer-Hermann, M., Deutsch, A., and Or-Guil, M. Recycling Probability and Dynamical Properties of Germinal Center Reactions. *J. Theor. Biol.* **210**, 265–285 (2001).

6. Batista, F. and Neuberger, M. Affinity dependencs of the B cell response to antigen: A threshold, a ceiling and the importance of off-rate. *Immunity* **8**, 751–759 (1998).

7. Randall, T., Parkhouse, R., and Corley, R. J chain synthesis and secretion of hexameric igm is differentially regulated by lipopolysaccharide and interleukin 5. *Proc. Natl. Acad. Sci. USA* **89**, 962–966 (1992).

8. Victora, G. D., Schwickert, T. A., Fooksman, D. R., Kamphorst, A. O., Meyer-Hermann, M., Dustin, M. L., and Nussenzweig, M. C. Germinal center dynamics revealed by multi-photon microscopy with a photoactivatable fluorescent reporter. *Cell* **143**, 592–605 (2010).

9. Gitlin, A. D., Shulman, Z., and Nussenzweig, M. C. Clonal selection in the germinal centre by regulated proliferation and hypermutation. *Nature* **509**, 637–640 (2014).

10. Berek, C. and Milstein, C. Mutation drift and repertoire shift in the maturation of the immune response. *Immunol. Rev.* **96**, 23–41 (1987).

11. Nossal, G. The molecular and cellular basis of affinity maturation in the antibody response. *Cell* **68**, 1–2 (1992).

12. Toellner, K. M., Jenkinson, W. E., Taylor, D. R., Khan, M., Sze, D. M. Y., Sansom, D. M., Vinuesa, C. G., and MacLennan, I. C. M. Low-level Hypermutation in T Cell-independent Germinal Centers Compared with High Mutation Rates Associated with T Cell-dependent Germinal Centers. *J. Exp. Med.* **195**, 383–389 (2002).

13. Chaturvedi, A., Martz, R., Dorward, D., Waisberg, M., and Pierce, S. K. Endocytosed BCRs sequentially regulate MAPK and Akt signaling pathways from intracellular compartments. *Nat. Immunol.* **12**, 1119–1126 (2011).

14. Omori, S. A., Cato, M. H., Anzelon-Mills, A., Puri, K. D., Shapiro-Shelef, M., Calame, K., and Richert, R. C. Regulation of Class-Switch Recombination and Plasma Cell Differentiation by Phosphatidylinositol 3-Kinase Signaling. *Immunity* **25**, 545–557 (2006).

15. Dustin, M. and Meyer-Hermann, M. Antigen feast or famine. *Science* **335**, 408–409 (2012).

16. Thaunat, O., Granja, A. G., Barral, P., Filby, A., Montaner, B., Collinson, L., Nartinez-Martin, N., Harwood, N. E., Bruckbauer, A., and Batista, F. D. The asymmetric segregation of polarized antigen on B cell division shapes presentation capacity. *Science* **335**, 475–479 (2012).

17. Schwickert, T. A., Lindquist, R. L., Schakhar, G., Livshits, G., Skokos, D., Kosco-Vilbois, M. H., Dustin, M. L., and Nussenzweig, M. C. In vivo imaging of germinal centres reveals a dynamic open structure. *Nature* **446**, 83–87 (2007).

18. Wang, P., Chang-ming, S., Qi, H., and Lan, Y.-h. A stochastic model of the germinal center integrating local antigen competition, individualistic T-B interactions, and B cell receptor signaling. *J Immunol* **197**, 1169–1182 (2016).

19. Papa, I., Saliba, D., Ponzoni, M., and Bustamante, S. e. a. Tfh-derived dopamine accelerates productive synapses in germinal centres. *Nature* **547**, 318–323 (2017).

20. Allen, C. D., Okada, T., Tang, H. L., and Cyster, J. G. Imaging of germinal center selection events during affinity maturation. *Science* **315**, 528–531 (2007).

21. Miller, M., Wei, S., Parker, I., and Cahalan, M. Two-photon imaging of lymphocyte motility and antigen response in intact lymph node. *Science* **296**, 1869–73 (2002).
